# Ascovirus P64 Homologs: A Novel Family of Large Cationic Proteins That Condense Viral Genomic DNA for Encapsidation

**DOI:** 10.3390/biology7030044

**Published:** 2018-09-11

**Authors:** Dennis K. Bideshi, Tatsinda Spears, Heba A. H. Zaghloul, Yeping Tan, Yves Bigot, Brian A. Federici

**Affiliations:** 1 Department of Biological Sciences, California Baptist University, Magnolia Avenue, Riverside, CA 92504, USA; 2 Department of Entomology, University of California, Riverside, CA 92521, USA; yeping747@163.com (Y.T.); brian.federici@ucr.edu (B.A.F.); 3 Graduate Program in Cell, Molecular and Developmental Biology, and Microbiology, University of California, Riverside, CA 92521, USA; tatsinda.spears@gmail.com (T.S.); hzagh001@ucr.edu (H.A.H.Z.); 4 UMR CNRS7247, Centre INRA Val de Loire, 37380 Nouzilly, France; yves.bigot@inra.fr

**Keywords:** insect viruses, ascovirus, SfAV-1a, P64 homologs, basic proteins, DNA condensation, encapsidation

## Abstract

Eukaryotic dsDNA viruses use small basic protamine-like proteins or histones, typically <15 kDa, to condense and encapsidate their genomic (g)DNAs during virogenesis. Ascoviruses are large dsDNA (~100–200 kbp) viruses that are pathogenic to lepidopteran larvae. Little is known about the molecular basis for condensation and encapsidation of their gDNAs. Previous proteomic analysis showed that *Spodoptera frugiperda ascovirus* (SfAV-1a) virions contain a large unique DNA-binding protein (P64; 64 kDa, *pI* = 12.2) with a novel architecture proposed to condense its gDNA. Here we used physical, biochemical, and transmission electron microscopy techniques to demonstrate that P64’s basic C-terminal domain condenses SfAV-1a gDNA. Moreover, we demonstrate that only P64 homologs in other ascovirus virions are unique in stably binding DNA. As similar protein families or subfamilies were not identified in extensive database searches, our collective data suggest that ascovirus P64 homologs comprise a novel family of atypical large viral gDNA condensing proteins.

## 1. Introduction

The condensation and packaging of viral genomes into capsids are essential steps in virogenesis [1,2,3,4]. In particular, virus genome condensation is largely mediated by neutralization of the negative electrostatic charge on the phosphate backbone of nucleic acids by cationic species to promote efficient encapsidation. Viruses utilize a variety of strategies to neutralize phosphate anions in their genomes, including sequestration of divalent cations and polyvalent polyamine cations such as spermine and spermidine, or small basic proteins rich in arginine and lysine that bind nucleic acids with high affinity [5,6]. Most notable among the latter are protamine-like proteins and histones that are ubiquitous in the nucleus. Histones play essential epigenetic roles, but primarily function in condensing and packaging chromosomal DNA into nucleosomes [7], a comparable function pirated by several DNA and RNA viruses, such as, respectively, polyomaviruses, herpes viruses [8,9,10,11,12], and retroviruses [13,14].

Although histones are abundant in the nucleus, they do not appear to be involved in the condensation and packaging of most virus genomes. Instead, viruses such as the hepatitis B virus (HBV) and baculoviruses encode small cationic proteins that function similar to histones in viral biology. For example, the *Autographa californica* multiple nucleopolyhedrovirus (AcMNPV) encodes a basic protamine-like protein, P6.9 (6.9 kDa), rich in arginine and serine, that condenses viral gDNA for encapsidation [15,16,17]. Prior to virion assembly, P6.9 is phosphorylated to prevent its binding to gDNA, but is subsequently dephosphorylated to accommodate condensation and packaging of the virus genome. Upon infection of host cells by AcMNPV virions, P6.9 is rephosphorylated by a capsid-associated kinase to liberate viral gDNA through electrostatic repulsion. The mechanism involved in AcMNPV’s gDNA condensation and release is apparently conserved among baculoviruses based on the presence of *p69* gene homologs in at least 50 known genomes of members of the *Baculoviridae* [17,18].

Like baculoviruses, ascoviruses are large dsDNA viruses that are primarily pathogenic to lepidopterans [19,20]. In comparison to baculoviruses, very little is known about the molecular biology of ascoviruses, and in particular, the mechanism involved in condensation and encapsidation of ascovirus gDNAs. Small protamine-like proteins such as P6.9 are not encoded by ascoviruses, and histones have not been detected in the virion proteomic profile of the type species, *Spodoptera frugiperda ascovirus* (SfAV-1a) [21]. However, we have shown previously that SfAV-1a virions contain at least 21 structural proteins of which only P64 (64 kDa) binds DNA stably in Southwestern assays [21,22]. P64 has a novel bipartite architecture not known to occur together in other proteins as it contains four copies of a virus-specific 2-cysteine adaptor (vs2C-ad) motif (residues 1–219; pfam08793; cl07414) with an intervening stretch of basic amino acids (95-RGTSPSRRSRSRSMSPRRRASPARRR-112) between two vs2C-ad in the N-terminal domain (residues 1–263), and 14 tandem repeats of an arginine/serine-rich motif [SPSQRRSTS(V/K)(A/S)RR] in the C-terminal domain (residues 279–455) [22]. The cationic property of P64 (*pI* = 12.2) and its marked abundance together with the (i) early expression of its corresponding gene (ORF048) [23], (ii) absence of histones and protamines in the virion [21], and (iii) its progressive localization from the virogenic stroma into the virion core [22], suggested that it plays an essential role in condensing the SfAV-1a genome for encapsidation. However, no direct evidence has been provided to demonstrate that P64 condenses SfAV-1a gDNA. Here we show that P64 and its domains and motifs, when assayed independently, condense SfAV-1a gDNA. We also show that P64 homologs in other ascoviruses are also one of the two most abundant proteins (the other being capsid protein) in their respective virions, and are the only known structural proteins that bind DNA in Southwestern assays. Based on our collective data, and considering that no other known proteins are characterized by a bipartite architecture composed of the vs2C-ad motif in the N-terminal domain and multiple tandem repeats of the serine/arginine-rich [SPSQRRSTS(V/K)(A/S)RR] basic motif in the C-terminal domain, we propose that P64 and its homologs comprise a novel family of atypical large basic proteins that condense ascovirus gDNA for encapsidation.

## 2. Materials and Methods

### 2.1. Virion Purification

To prepare a stock of ascovirus virions, *Spodoptera exigua* larvae were infected by puncturing the abdomen of late third-early fourth instars with a minutin pin that was dipped in a suspension of viral vesicles (~1 × 10^8^ virion vesicles/mL), as described previously [23]. Nine days post-infection, the hemolymph of infected larvae was collected in ice-cold phosphate-buffered saline (PBS, pH 7.4) with 1% glutathione. Infected hemolymph was sonicated for 30 s at 50% duty cycle using the Ultrasonic Homogenizer 4710 series (Cole-Parmer Instruments, Vernon Hills, IL, USA) then spun at 1200 × *g* using a TS-5.1-500 rotor in the Allegra 25R centrifuge (Beckman-Coulter, Inc., Brea, CA, USA) for 10 min at 4 °C. The supernatant was layered on top of a sucrose gradient (20–55%) at 4 °C and centrifuged for 1 h at 104,000 × *g* using a Beckman SW28 rotor. The band containing virions was collected and washed in ice-cold PBS, and spun at 104,000 × *g* for 1 h at 4 °C. The SfAV-1a virion pellet was resuspended in PBS and stored at −80 °C.

### 2.2. Isolation of Recombinant 6x-Histidine-Tagged Proteins

Recombinant 6x-histidine-tagged P64 (rP64) was produced using the Bac-to-Bac (Invitrogen, Carlsbad, CA, USA) Baculovirus expression kit as previously described [22]. The vs2C-ad domain in the amino-terminal (rN-term) and the basic repeats in carboxy-terminal (rC-term) of P64 were expressed with the 6x-histidine tag on the amino-terminus of each recombinant and purified in a similar manner as rP64 [22]. SfAV-1a gDNA was purified with DNAzol (Invitrogen) according to the manufacturer’s instructions and used for PCR. The rN-term included the first 219 amino acids of P64 (SfAV1a ORF048) [24] and was amplified by PCR using the primer pair P64forward 5′-CGCGGATCCATGGCGTCAAAACGTAAA-3′ and N1reverse 5′-ATACTCGAGGGCGCCGTGACACATGCT-3′. Amino acids 265 to 565 of P64 were included in the rC-term and amplified by PCR using the primer pair Cforward 5′-AGAGGATCCAGCACGAGTCGTTCCAAG-3′ and P64reverse 5′-CCGCTCGAGATCCTTCGACGATCAGGT-3′.

### 2.3. SDS-PAGE, Western and Southwestern Blotting

Protein concentrations were determined by the method of Bradford [25]. Virion proteins from different species of AV were fractionated by SDS-PAGE, blotted on polyvinylidene difluoride (PVDF) membranes (GE Osmonics, Minnetonka, MN, USA) and analyzed by Western blot analysis using an anti-P64 antibody [22]. Southwestern analysis was also performed, as described previously [22], with minor modifications, i.e., 15 ng of DIG-labeled SfAV1a ORF60 dsDNA [24] was suspended in 2 mL of DNA binding buffer and incubated with the blot for 2 h at 25 °C in a sealed plastic pouch. After washing and blocking, the membrane was incubated with anti-DIG antibody for 2.5 h and detection of labeled DNA was performed using the NBT/BCIP reagents, according to manufacturer’s protocol (Roche Diagnostic, Noblesville, IN, USA). The DIG-labeled ORF60 DNA was produced by amplifying the 237 bp gene from SfAV1a gDNA with the forward primer 5′-gagctcTTGATCTGATCATGATAAAACGTATAC-3′ and reverse primer 5′-atcgatTCATCATCCCTCAGTTGGATAAAACTT-3′. The QIAquick PCR Purification kit (QIAGEN) was used to purify the amplicon for labeling with the DIG Oligonucleotide 3’-End Labeling kit, 2nd Generation (Roche Diagnostics, Noblesville, IN, USA).

Southwestern analysis was also performed with purified rP64, rN-term, and rC-term (~2 μg, respectively), and peptides (0–8 μg) containing the basic motifs in the N-terminal (95-RGTSPSRRSRSRSMSPRRRASPARRR-112) and C-terminal (SPSQRRSTSVARR) (EZBiolabs, Inc., Carmel, CA, USA), and 15 ng of SfAV-1a or phage λ genomic (g)DNA, as described previously [22]. After washing and blocking, the membrane was incubated with anti-DIG antibody for 2.5 h and detection of labeled DNA was performed using the NBT/BCIP reagents, according to manufacturer’s protocol (Roche).

### 2.4. DNA-Protein Complex Aggregation

DNA-binding reactions and electrophoretic mobility shift assays (EMSA) were performed as described previously [22] with minor modifications, i.e., purified rP64, rN-term, rC-term (~2 μg/mL, respectively), or peptides (0–8 μg/mL) containing the basic motifs in the N-terminal (95-RGTSPSRRSRSRSMSPRRRASPARRR-112) and C-terminal (SPSQRRSTSVARR) (EZBiolabs, Inc., Carmel, CA, USA), and 15 ng of SfAV-1a genomic (g)DNA, or phage λ (g)DNA which was used a non-specific dsDNA control. After incubation, protein-DNA complexes were pelleted at 16,300 g for 30 min at 25 °C. Supernatants were transferred to new tubes and mixed with 1.5 μL glycerol. The remaining pellets were dissolved in 10 μL of 0.1 X TE and 1 μL glycerol. Supernatant and pellet fractions for each protein-DNA combination were resolved by electrophoresis (100 volts, 1 h) in a 0.7% agarose gel containing GelRed (Phenix Research Products, Candler, NC, USA).

### 2.5. Transmission Electron Microscopy

SfAV1a gDNA-protein complexes were prepared according to the DNA-protein complex aggregation method described above. After centrifugation and separation of supernatant and pellet fractions, resuspended pellets or supernatant from the SfAV1a gDNA and BSA-gDNA reactions (negative controls) were adsorbed onto discharged carbon-coated formvar grids, as described previously [26,27]. Samples were first placed on parafilm. A grid was touched to the surface of the droplet of sample for 90 s, then lifted and touched to the surface of a drop of 0.25 M ammonium acetate for 40 s, then lifted and touched to the surface of a drop of 4% uranyl acetate in 50% ethanol. Grids were touched to a drop of 95% ethanol twice and flicked to remove the excess ethanol each time then allowed to dry. Duplicate samples were examined by TEM (FEI Tecnai), and representative foci of 11–15 spots screened were photographed with a Gatan US 1000 camera.

### 2.6. Database Searches

The predicted amino acid sequence of P64 homologs was analyzed using various online programs, including BLAST at the NCBI website (http://www.ncbi.nlm.nih.gov/), Scratch protein predictor (http://www.ics.uci.edu/~baldig/scratch/), pfam (http://pfam.janelia.org/search), MEME (http://meme.nbcr.net/meme/intro.html), and (http://supfam.org/SUPERFAMILY/) to identify gene homologues, orthologues, conserved domains, motifs, and secondary structure, and to identify possible families and superfamilies of proteins to which the SfAV-1a P64 homologs belong.

## 3. Results

### 3.1. P64 and its N-Terminal and C-Terminal Domains Condense and Precipitate SfAV-1a gDNA

**Figure 1:**
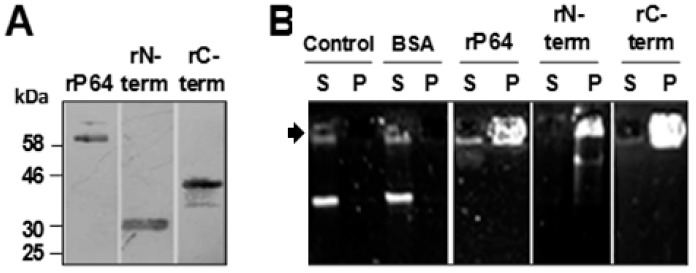
P64 and its N-terminal (N-term) and C-terminal (C-term) domains condense *Spodoptera frugiperda ascovirus-1a* genomic (g)DNA. (**A**) SDS-PAGE showing purified 6x-histidine tagged proteins (rP64, rN-term, rC-term) used in the assays. (**B**) Examples of results of electrophoretic mobility shift assays (EMSA) showing the presence of SfAV-1a gDNA in supernatants (S) or pellets (P) after centrifugation following incubation without protein or with bovine serum albumin (BSA), rP64, rN-term and rC-term; ~2 μg of each protein and 15 ng of gDNA were used in each assay. Arrowhead indicates position of wells in agarose gels.

To demonstrate whether P64 and its *N*-terminal (*N*-term; residues 1–219) and *C*-terminal (*C*-term; residues 265–565) domains could independently bind, condense, and precipitate SfAV-1a gDNA in vitro, purified recombinant 6x-histidine (6x-his) tagged protein, rP64, and 6x-his tagged rN-term and rC-term peptides (Figure 1A) were incubated with SfAV-1a gDNA. Stable protein-DNA complexes were collected by centrifugation and their presence in pellets and supernatants were analyzed by agarose gel electrophoresis. Following centrifugation of the mixtures, gDNA was observed only in pellets but not in supernatants, a result not observed with a BSA (bovine serum albumin) control (Figure 1B).

### 3.2. Basic Motifs in P64’s N-Terminal and C-Terminal Domains Bind DNA Non-Specifically

**Figure 2:**
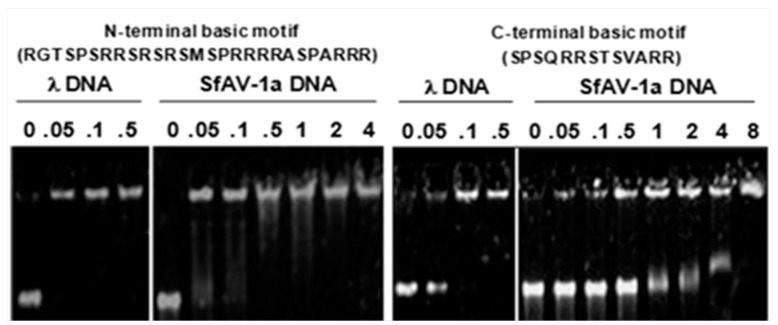
Electrophoretic mobility shift assays showing that synthetic peptides (0.5–8 μg/mL) of basic motifs found in the N-terminal and C-terminal domains of P64 can retard mobility of phage λ and SfAV-1a gDNAs.

P64 contains a single copy of a basic motif (RGTSPSRRSRSRSMSPRRRRASPARRR) in its N-terminal domain, whereas 14 tandem repeats of another basic motif (SPSQRRSTS[V/K][A/S]RR) are present in the C-terminal domain [22]. To determine whether synthetic peptides of these motifs could independently bind DNA, various amounts (0.5–8 μg/mL) of each peptide was mixed with either SfAV-1a or phage λ gDNAs, and peptide-DNA complexes were analyzed by electrophoretic mobility shift assays. Both peptides retarded the mobility of phage λ and SfAV-1a gDNAs (Figure 2) indicating their non-specific intermolecular interactions with DNA.

### 3.3. Transmission Electron Microscopy (TEM) Demonstrates P64 and its C-terminal Domain SfAV-1a Condense gDNA

**Figure 3:**
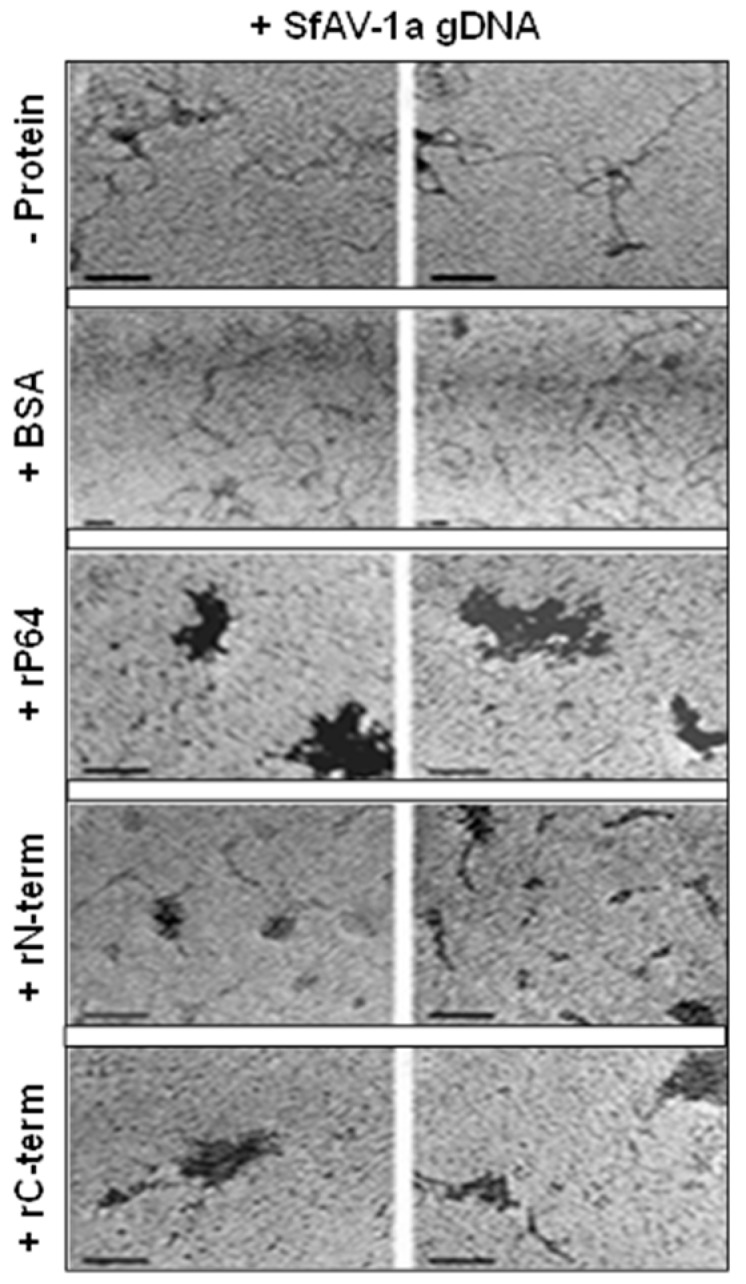
Examples of transmission electron micrographs demonstrating that rP64 and it rC-terminal domain (rC-term) alone precipitate and condense SfAV-1a genomic (g)DNA in vitro. Significantly less extensive precipitation and condensation occurred with the rN-terminal (rN-term) domain of P64. Controls included gDNA without protein (-protein) or with bovine serum albumin (BSA). Bar = 50 nm.

The ability of rP64, rN-term and rC-term to condense SfAV-1a gDNA was further analyzed by TEM of negative stained samples of protein-gDNA complexes using well established methods [26]. TEM revealed uncondensed gDNA either in the absence or presence of BSA, whereas rP64 and rC-term condensed gDNA extensively, forming dense foci on the grids (Figure 3). The appearance of the heterogeneous aggregates was similar to those observed in other studies using these methods to specifically demonstrate protein-induced DNA condensation [28]. Interestingly, significantly less extensive condensates were observed with the rN-term domain harboring the four repeats of the virus-specific two-cysteine adaptor (vs2C-ad) motif with its intervening basic stretch of amino acids that was shown to bind phage λ and SfAV-1a gDNAs independently (Figure 2).

### 3.4. P64 Homologs Are Abundant DNA-Binding Proteins in Virions of Ascoviruses

**Figure 4:**
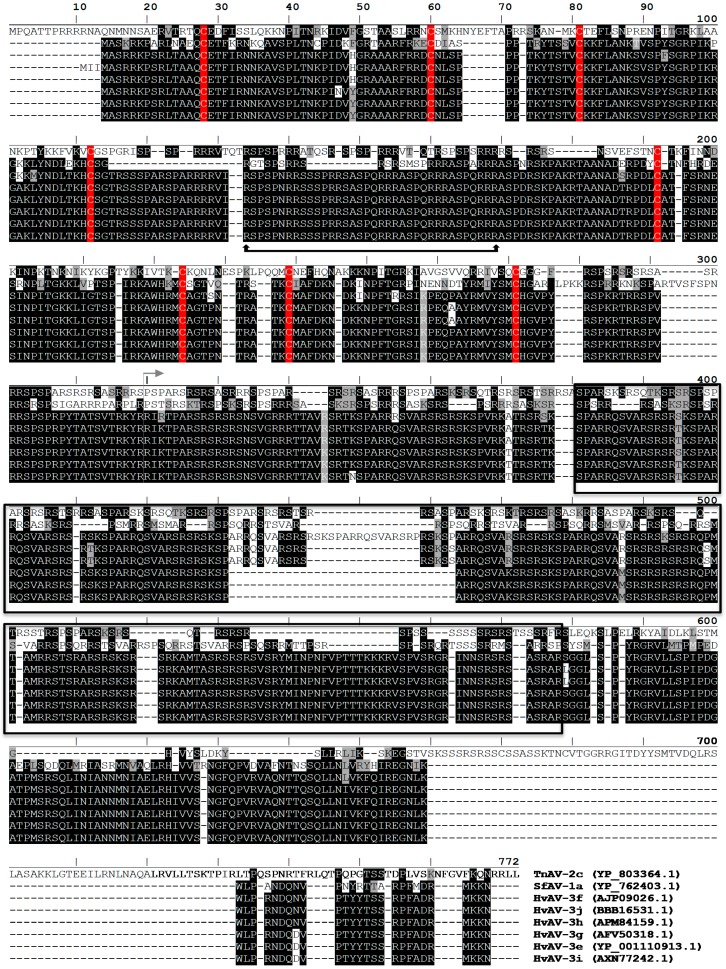
Alignment of ascovirus (AV) P64 homologs encoded by *Trichoplusia ni* AV-2c (TnAV-2c), *Spodoptera frugiperda AV-1a* (SfAV-1a), and various isolates of *Heliothis virescens* AV (HvAV-3e, -3f, -3g, -3h, -3i, -3j). GenBank accession numbers in parentheses, conserved cysteines (highlighted red) in the virus-specific 2-cysteine adaptor (vs2C-ad) motif and intervening stretch of basic residues (bold underline with vertical arrows) in the N-terminal domain, and arginine/serine rich repeats in the C-terminal domain (boxed, black line) are shown; the N-terminal and C-terminal domains are delineated (grey horizontal arrow) based on the SfAV-1a’s P64 protein (Tan et al., 2009 [22]).

**Figure 5:**
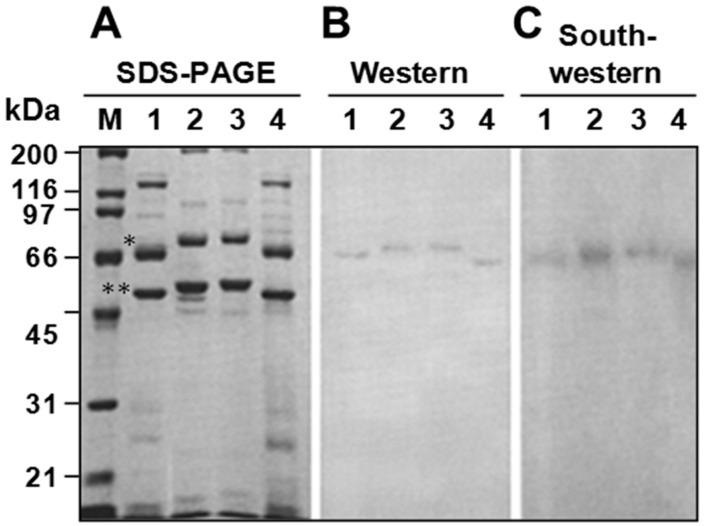
Ascovirus SfAV-1a P64 homologues are the only major virion proteins that bind DNA. (**A**) SDS-PAGE profiles of virion proteins of SfAV-1a (lane 1), *Heliothis virescens* AV-3a (lane 2), *Trichoplusia ni* AV-6a (lane 3), and *Spodoptera exigua* AV-7a (lane 4) probed with an anti-P64 antibody (**B**), or labeled dsDNA (**C**). The most abundant virion proteins are the P64 homologs (*) and the major capsid protein, MCP (**). M, molecular mass standards; kDa, kilodaltons.

In database searches, we identified P64 homologs with a similar 2-domain architecture (Figure 4); these homologs were unique to ascoviruses. Virion protein profiling by SDS-PAGE and Western blot using an anti-P64 antibody (Figure 5) revealed that these homologs, together with the major capsid protein (MCP) [21,22], were the most abundant virion proteins in all ascovirus species included in the survey. Moreover, the P64 homologs were the only virion proteins that stably bound DNA, at least in the constraint of our Southwestern assay (Figure 5).

### 3.5. Ascovirus P64 Homologs Comprise a Novel Family of Unusually Large Cationic Proteins That Condense Viral DNA for Encapsidation

In our extensive database searches, we did not identify proteins that contained similar distinct N-terminal and C-terminal domains and motifs that conformed to the architecture of the ascovirus P64 homologs.

## 4. Discussion

In the present study, we demonstrate that cationic P64 and its structural domains and basic motifs independently bind, precipitate, and condense SfAV-1a gDNA. As P64 (i) is a structural component of the virogenic stroma [22]; (ii) is progressively incorporated into the virion core [22]; (iii) has conserved homologs in other ascovirus species that are the only known DNA-binding protein in their respective virions (Figure 4 and Figure 5); and (iv) genes coding for small basic proteins are absent in ascovirus genomes [29], the cumulative data indicate that P64 and its ascovirus homologs comprise a novel family of atypical large viral gDNA condensing proteins, not known to occur in other viruses. Indeed, to our knowledge the P64 homologs represent the largest known virion structural protein with this essential function.

Whereas arguably it is expected that the highly basic arginine/serine-rich C-terminal domain of P64 is primarily involved in DNA condensation, the role of the N-terminal domain is less clear, as it precipitates SfAV-1a gDNA comparable to the C-terminal domain (Figure 1B), likely because of the presence of the arginine-rich intervening residues (Figure 2), yet it does not appear to condense DNA extensively as the C-terminal peptide, as determined by transmission electron microscopy (Figure 3). It is possible that the single intervening stretch rich in arginine found between the two pairs of virus-specific 2-cysteine adaptor (vs2C-ad) in the N-terminal domain is not exposed sufficiently under its native conformation to effectively interact with DNA, and as such, it may not have a natural role in directly interacting with gDNA. Alternatively, the P64 N-terminal domain could have a dual function that includes both protein-protein and protein-DNA interactions. Regardless, at present, specific function(s) of the N-terminal vs2C-ad motif remains unknown [30], but interestingly, the vs2C-ad has been found fused to OTU/A20-like peptidases and serine/threonine protein kinases, primarily in proteins encoded by members of the nucleo-cytoplasmic large DNA viruses (NCLDV), which includes ascoviruses [30,31]. As such, interactions with the putative serine/threonine kinase and CTD (carboxy-terminal domain)-like phosphatase identified in the SfAV-1a virion proteome [21] could facilitate phosphorylation/dephosphorylation of P64 to allow for, respectively, uncoupling and coupling of the protein from gDNA. This seems likely, as we have demonstrated previously that nascent P64 is heavily phosphorylated but exists in an unphosphorylated form in the virion [22]. However, further studies are required to determine whether these three proteins function as a unit in condensing and releasing SfAV-1a gDNA in the virion.

With regard to the evolutionary origin of the P64 family of proteins, comparative analysis of the conserved motifs of homologs in SfAV-1a, TnAV-2c, and the HvAV isolates (Figure 4) suggests that putative orthologs could contain significant variations in the number and repeats of the vs2C-ad and basic SPSQRRSTS(V/K)(A/S)RR motifs. Therefore, in an attempt to identify candidates related to SfAV-1a P64 in more distantly related viruses, databases were mined to identify basic proteins with (i) an apparent *pI* of >9; (ii) one or several vs2C-ad motifs; and (iii) one or several kinds of motifs repeated in tandem and rich in arginine and serine residues. Two groups of proteins matched these criteria and were specific to members of *Ascoviridae* and *Iridoviridae*, which interestingly, are considered to be ancestral to AVs [29,32]. No match was found in other Megavirales [33]. The first group of proteins is a priori restricted to all members of the two genera of invertebrate iridoviruses (IIV), the *Iridovirus* and the *Chloriridovirus*, and two representatives of a single species of unclassified crustacean iridoviruses [34,35]. It is comprised of orthologs encoded by the Chilo iridescent virus 6 (CIV6 [IIV6], ORF176R) (Supplemental Figure S1a). Members of this group have homogeneous sequence features consisting of about 215 residues and contain one vs2C-ad motif at the N-terminal end to which is juxtaposed a basic motif repeated several times in tandem. In the central region, they also contain two 38-residue motifs separated by a conserved 14-residue linker. The protein size and sequence conservation in this group of CIV6 ORF176R orthologs were not consistent with the organizational properties of the SfAV-1a P64 orthologs. The sequence features of the second group were consistent with those of the SfAV-1a P64 orthologs (Supplemental Figure S1b). Relatives were found in *Diadromus pulchellus toursvirus* (DpTV, previously DpAV4; ORF008) [19], in two IIVs of the genus *Iridovirus* (IIV6, ORF232R; IIV31 ORF015R), in the two unclassified crustacean iridoviruses [34,35], and in vertebrate iridoviruses belonging to the *Ranavirus* genus. No relatives were found in genomes of IIV3 [36], IIV22 [37], IIV25 [38], IIV30 [39], and IIV31 [40]. As expected, SfAV-1a P64 orthologs in these viruses were found to contain from 1 to 6 repeats of the vs2C-ad motif, and at least two types of tandemly repeated basic motif located between the vs2C-ad and/or in the central region of these proteins. The variation in sequence, organization, and features of SfAV-1a P64 orthologs indicate that these putatively distantly related proteins (Supplemental Figure S1a–c) evolved rapidly in a manner that hampered their accurate alignment in silico, which as a consequence prevented meaningful inferences regarding their evolution. Therefore, the origin of the P64 family of virus gDNA condensing proteins remains elusive at present.

Finally, although little has been published on the molecular biology of ascoviruses, in comparison to other insect viruses such as baculoviruses, it is clear that these entomopathogens are unique among all known viruses with regard to their ultrastructure, pathobiology, and cytopathology [19,20]. For example, ascovirus virions exhibit an atypical reticulate pattern when negatively stained and examined by electron microscopy. Ascovirus replication is even more atypical. After infection, in a process resembling apoptosis, the nucleus lyses and the cell is cleaved into numerous vesicles that disperse to the hemolymph. Viral replication continues as these vesicles circulate in the hemolymph, generating hundreds of virions per vesicle, which serve as reservoirs for horizontal transmission. When laying eggs in infected caterpillars, female parasitoid wasps acquire viral vesicles and virions on the ovipositor, and vector these to new hosts during subsequent oviposition events [20,41,42]. In SfAV-1a, the apoptosis-like process is initiated by a virus-coded executioner caspase, another distinctive feature of ascoviruses, as no other viruses are known to encode functional caspases [24,43]. In this regard, based on these unique features, it is perhaps also not surprising that ascoviruses utilize the novel P64 family of basic proteins to sequester their gDNAs for encapsidation during virogenesis.

## 5. Conclusions

Our study shows that, invariably, ascoviruses encode a novel basic protein composed of a bipartite architecture in which the N-terminal domain contains multiple copies of a virus-specific 2-cysteine adaptor (vs2C-ad) motif (pfam08793; cl07414) with an intervening stretch of basic amino acids between two vs2C-ad, and multiple tandem repeats of an arginine/serine-rich motif in the C-terminal domain. This protein, first identified and reported in SfAV-1a and named P64 [22,24], and its homologs in other ascoviruses are the predominant virion structural proteins shown to stably bind, condense, and precipitate dsDNA. Finally, as the P64 homologs are unique to ascoviruses, and their distinct structural architecture is absent in other known proteins, we propose that the P64 homologs constitute a novel family of unusually large viral proteins essential for viral genome condensation and encapsidation during virogenesis.

## Supplementary Materials

The following are available online at http://www.mdpi.com/2079-7737/7/3/44/s1, Figure S1a: Amino acid sequences of Chilo iridescent virus 6 (CIV 6, IIV6)-178R ORF orthologs in classified (a) and unclassified (b) invertebrate iridescent viruses (IIV); Figure S1b: Amino acid sequences of SfAV1a-048R ORF orthologs; Figure S1c: Proteins having the potential to function like SfAV1a P64 protein in some invertebrate iridoviruses (IIV) and members of Lymphocystivirus and Megalocytivirus.

## References

[1] Chelikan V., Rajan T., Kondabagil K. (2014). Revisiting the genome packaging in viruses with lessons from the “Giants”. Virology.

[2] Cuervo A., Dauden M.I., Carroscosa J.L. (2013). Nucleic acid packaging in viruses. Subcell. Biochem..

[3] Lieberman P.M. (2008). Chromatin organization and virus gene expression. J. Cell. Physiol..

[4] Locker R.C., Fuller S.D., Harvey S.C. (2007). DNA organization and thermodynamics during viral packing. Biophys. J..

[5] Brewer L.R., Corzett M.R., Balhorn R. (1999). Protamine-induced condensation and decondensation of small DNA molecules. Science.

[6] Koltover I., Wagner K., Safinya C.R. (2000). DNA condensation in two dimensions. Proc. Natl. Acad. Sci. USA.

[7] Bartova E., Krejci J., Harnicarova A., Galiova G., Kozubek S. (2008). Histone modifications and nuclear architecture: A review. J. Histochem. Cytochem..

[8] Bulach D.M., Kumar C.A., Zaia A., Liang B., Tribe D.E. (1999). Group II nucleopolyhedrovirus subgroups revealed by phylogenetic analysis of polyhedron and DNA polymerase gene sequences. J. Invertebr. Pathol..

[9] Campos E.I., Reinberg D. (2009). Histones: Annotating chromatin. Annu. Rev. Genet..

[10] La Bella F., Vesco C. (1980). Late modifications of simian virus 40 chromatin during the lytic cycle occur in an immature form of virion. J. Virol..

[11] Milavetz B. (2004). Hyperacetylation and differential deacetylation of histones H4 and H3 define two distinct classes of acetylated SV40 chromosome early in infection. Virology.

[12] Imai K., Kamio N., Cueno M.E., Saito Y., Inoue H., Saito I., Ochiai K. (2014). Role of the histone H3 lysine 9 methyltransferase Suv39 h1 in maintaining Epstein-Barr virus latency in B95-8 cells. FEBS J..

[13] Jeng M.Y., Ali I., Ott M. (2015). Manipulation of the host protein acetylation network by human immunodeficiency virus type 1. Crit. Rev. Biochem. Mol. Biol..

[14] Segura M.M., Garnier A., Di Falco M.R., Whisell G., Meneses-Acosta A., Archand N., Kamen A. (2008). Identification of host proteins associated with retroviral vector particles by proteonomic analysis of highly purified vector preparations. J. Virol..

[15] Wilson M.E., Mainprizw T.H., Friesen P.D., Miller L.K. (1987). Location, transcription, and sequence of a baculovirus gene encoding a small arginine-rich polypeptide. J. Virol..

[16] Wilson M.E., Price K.H. (1988). Association of *Autographa californica* nuclear polyhedrosis virus with nuclear matrix. Virology.

[17] Wang M., Tuladhar E., Shen S., Wang H., van Oers M.M., Vlak J.M., Westenberg M. (2010). Specificity of baculovirus P6.9 basic DNA-binding proteins and critical role of the C terminus in virion formation. J. Virol..

[18] Van Oers M.M., Vlak J.M. (2007). Baculovirus genomics. Curr. Drug Targets.

[19] Asgari S., Bideshi D.K., Bigot Y., Cheng X.W. (2017). ICTV Virus Taxonomy Profile: Ascoviridae. ICTV Report Consortium. J. Gen. Virol..

[20] Bideshi D.K., Bigot Y., Federici B.A., Spears T., Asgari S., Johnson K.N. (2010). Ascoviruses. Insect Virology.

[21] Tan Y., Bideshi D.K., Johnson J.J., Bigot Y., Federici B.A. (2008). Proteomic analysis of the *Spodoptera frigiperda* ascovirus 1a virion reveals 21 proteins. J. Gen. Virol..

[22] Tan Y., Spears T., Bideshi D.K., Johnson J.J., Hice R., Bigot Y., Federici B.A. (2009). P64, a novel major virion DNA-binding protein potentially involved in condensing the *Spodoptera frugiperda ascovirus 1a* genome. J. Virol..

[23] Zaghloul H.A.H., Hice R., Arensburger P., Federici B.A. Transcriptome analysis of the *Spodoptera frugiperda ascoviruis* in vivo provides insights into how its apoptosis inhibitors and caspase promote increased synthesis of viral vesicles and virion progeny. J. Virol..

[24] Bideshi D.K., Demattei M.V., Rouleux-Bonnin F., Stasiak K., Tan Y., Bigot S., Bigot Y., Federici B.A. (2006). Genomic sequence of the *Spodoptera frugiperda ascovirus 1a*, an enveloped, double-stranded DNA insect virus that manipulates apoptosis for viral reproduction. J. Virol..

[25] Bradford M.M. (1976). A rapid and sensitive method for the quantitation of microgram quantities utilizing the principle of protein dye binding. Anal. Biochem..

[26] Vollenweider J., Sogo J.M., Koller T.A. (1975). Routine method for protein-free spreading of double and single-stranded nucleic acid molecules. Proc. Natl. Acad. Sci. USA.

[27] Black B.C., Center M.S. (1979). DNA-binding properties of the major core protein of adenovirus 2. Nucleic Acids Res..

[28] Yu H., Ren J., Qu X. (2007). Time-dependent DNA condensation induced by amyloid β-peptide. Biophys. J..

[29] Bigot Y., Renault S., Nicolas J., Moundras C., Demattei M.V., Samain S., Bideshi D.K., Federici B.A. (2009). Symbiotic virus at the evolutionary intersection of three types of large DNA viruses; iridoviruses, ascoviruses, and ichnoviruses. PLoS ONE.

[30] Iyer L.M., Balaji S., Koonin E.V., Aravind L. (2006). Evolutionary genomics of nucleo-cytoplamsmic viruses. Virus Res..

[31] Rodrigues R.A., Abrahão J.S., Drumond B.P., Kroon E.G. (2016). Giants among larges: How gigantism impacts giant virus entry into amoebae. Curr. Opin. Microbiol..

[32] Stasiak K., Renault S., Demattei M.V., Bigot Y., Federici B.A. (2003). Evidence for the evolution of ascoviruses from iridoviruses. J. Gen. Virol..

[33] Colson P., De Lamballerie X., Yutin N., Asgari S., Bigot Y., Bideshi D.K., Cheng X.W., Federici B.A., Van Etten J.L., Koonin E.V. (2013). “Megavirales”, a proposed new order for eukaryotic nucleocytoplasmic large DNA viruses. Arch. Virol..

[34] Qiu L., Chen M.M., Wan X.Y., Li C., Zhang Q.L., Wang R.Y., Cheng D.Y., Dong X., Yang B., Wang X.H. (2107). Characterization of a new member of *Iridoviridae*, Shrimp hemocyte iridescent virus (SHIV), found in white leg shrimp (*Litopenaeus vannamei*). Sci. Rep..

[35] Li F., Xu L., Yang F. (2017). Genomic characterization of a novel iridovirus from redclaw crayfish *Cherax quadricarinatus*: Evidence for a new genus within the family Iridoviridae. J. Gen. Virol..

[36] Delhon G., Tulman E.R., Afonso C.L., Lu Z., Becnel J.J., Moser B.A., Kutish G.F., Rock D.L. (2006). Genome of invertebrate iridescent virus type 3 (mosquito iridescent virus). J. Virol..

[37] Piégu B., Guizard S., Spears T., Cruaud C., Couloux A., Bideshi D.K., Federici B.A., Bigot Y. (2013). Complete genome sequence of invertebrate iridescent virus 22 isolated from a blackfly larva. J. Gen. Virol..

[38] Piégu B., Guizard S., Spears T., Cruaud C., Couloux A., Bideshi D.K., Federici B.A., Bigot Y. (2014). Complete genome sequence of an invertebrate iridovirus IIV-25 isolated from a blackfly larva. Arch. Virol..

[39] Piégu B., Guizard S., Spears T., Cruaud C., Couloux A., Bideshi D.K., Federici B.A., Bigot Y. (2014). Complete genome sequence of invertebrate iridovirus IIV30 isolated from the earworm, *Helicoverpa zea*. J. Invertebr. Pathol..

[40] Piégu B., Guizard S., Yeping T., Cruaud C., Asgari S., Bideshi D.K., Federici B.A., Bigot Y. (2014). Complete sequence of a crustacean iridoviris, IIV31, isolated from the pill bug, *Armadillidium vulgare*. J. Gen. Virol..

[41] Federici B.A. (1983). Enveloped double-stranded DNA insect virus with novel structure and cytopathology. Proc. Natl. Acad. Sci. USA.

[42] Federici B.A., Bideshi D.K., Tan Y., Spears T., Bigot Y. (2009). Ascoviruses: Superb manipulators of apoptosis for viral replication and transmission. Curr. Top. Microbiol. Immunol..

[43] Bideshi D.K., Tan Y., Bigot Y., Federici B.A. (2005). A viral caspase contributes to modified apoptosis for virus transmission. Genes Dev..

