# Supplementary material for: Ascovirus P64 Homologs: A Novel Family of Large Cationic Proteins That Condense Viral Genomic DNA for Encapsidation
Source: Biology (Basel). 2018 Sep 11;7(3):44. doi: 10.3390/biology7030044 (PMC6163548; doi:10.3390/biology7030044)
Supplement: Supplementary file 1 [file biology-07-00044-s001.pdf]

biology-348173_Supplementary material/bideshi et al.

Figure S1a: Amino acid sequences of Chilo iridescent virus 6 (CIV 6, IIV6)-178R ORF orthologs in classified (a) and unclassified (b) invertebrate iridescent viruses (IIV).

Conserved cysteine residues in vs2C-ad motifs are typed and underlined in red and, whereas the complete motif is highlighted in yellow. Motifs that are repeated in tandem and rich in arginine-serine residues are highlighted in gray. The two 38-residues motifs that are separated by a conserved 14-residues linkers are typed in blue.

a. Classified IIVs

>IIV3-100L ; pI = 10.17
MNDVHDCLVWVNDPYFHPISKRPIKYKGPTWRHYDKKCDLLGITGPKATKSPSRRTTRSP
SPSRRTTRSSPSRRTTRSSPSRRTTRSPSPSGRRKQGGPAVYCGNNALDEGLLDGSKVVG
TRYQCLQKGVAVGLNNPVLHHSPNYQPIVDAKIYCGTGSKLPASKLRFGTPTECMGKGYQ
IGQNKRFQQSGLQQGPYIWEEDGWYKIIVPKN

>IIV6-378R ; pI = 10.61
MTSKCSKWHEQPLINPLTNRKIKKNGPTYKELERECGPPPRRSSPRRSSPRRSPRRSSPR
RSSPRRSSPRRSSPRRSNQRIQLYCGNNARDEGLINGTKTLGTRYQCLKKGIGKGLNEPI
LKYNNDYEPIENVRIYCGNGALPNNKDRFGTRDECLRKGFAVGQKQKYIRDGGIQRGPIV
VEENGWYKAYLPR

>IIV9-085R ; pI = 10.71
MANKCVMWHLQPLVNPVTNRKIKRGGLVYQKLEQECGPPPSGSRRSPSPSRRRSPSPSRR
SSRRSPSPSRRSSRRSPSPSRRSLPPPQRQPELYCGNNSRERGLVDGTKVLGTRYQCLKK
GIGRGLKEPIFTWSDEYSPIEEVKVFCGNGDVLPQNKDRFGTRDECLRKGFAVGQHQKYT
RDGGIQRGPVVSEDRGWYKVYLPSALGPPVLGIRN

>IIV22-ORF138R ; pI = 11.45
MTSNCAKWHSQPLVNPVTNRKIKVGGPKYKELERECGPPPSRRSPSPSRRSRRSPSPSRR
SRRSPSPSRRSRRSPSPSRRSRRSPSPSRRSRRSPSPSRRSRRSPSPSRRPTEIYCGNNA
RDEGLINGSKILGTRYQCLKKGIGRGLHEPILSYTNDYSPIEEVKIFCGNGNVLPQNKDR
FGTRDECLRKGFAVGQNQKYTRDGDIQRTPVVSEDKGWYKVYLPSALGAPILRR

>IIV22A-136R ; pI = 11.02
MTSNCAKWHSQPLVNPVTNRKIKVGGPKYKELERKCGPPPSRRSRRSPSPSRRSRRSPSP
SRRSRRSPSPSRRSRRSPSPSSRRSPSPSRRPAEIYCGNNARDEGLINGTKILGTRYQCL
KKGIGRGLHEPILSYTNDYSPIEEVKIFCGNGNVLPQNKDRFGTRDECLRKGFAVGQNQK
YTRDGDIQRTPVVSEDKGWYKVYLPSALGAPILRR

>IIV25-136R ; pI = 11.37
MANKCAMWHLQPLVNPVTNRKIKRGGLVYQKLEQECGPPPSGSRRRSPPPSGRRSPSPSR
RRSPPPSGRRSPSPSRRRSPSPLRRSSSRRSPSPLRRSSSRRSPSPSRRSLPPQRQPELY
CGNNAREQGLLNGTKVLGTRYQCLKKGIGRGLNEPIFSYSDEYSPIEEVKIFCGNGNVVP
QNKDRFGTRDECLRKGFAVGQHQKYTRDGGIQRGPVVSEDRGWYKVYLPSALGPPVLGIR
N

>IIV30-135R ; pI = 11.02
MTSNCAKWHSQPLVNPVTNRKIKVGGPKYKELEQKCGPPPSRQSRRSPSPSRQSRRSPSP
SRQSRRSPPPSRQSRRSPSPSRRSRRSPSPSRQSRRSPSPSRRPTEIYCGNNARDEGLIN
GTKILGTRYQCLKKGIGRGLHEPILSYTNDYSPIEEVKIFCGNGNVLPQNKDRFGTRDEC
LRKGFAVGQNQKYTRDGDIQRTPVVSEDKGWYKVYLPSALGAPILRR

>IIV31-ORF130R ; pI = 10.43
MSKICKDWHEQPLVNPVTNRIIKKNGPTYRELEKKCGPPMTRRSSPRRSSPRRSSPRRSS

PRRSSPRRSSPRRSSPIRRNPETYCGNNAKDEGLTNGTKVLGNRFQCLRKGIGRGLNEPI
FTYSEEYEPIENIKVYCGNNANLPRDKDRFGTRDECLRKGFAVGQKQKYSRDGGVQKEPI
ITQDRGWYKVYVPRST

b. Unclassified IIVs

>117R Cherax quadricarinatus iridovirus - 117R ; pI = 11.70
MRVNENICQEFSNRPSVNPLTNRKIQKGKGVYNELKKKCDSLGCRVQSRSRSRSRSRRSS
RRRSSRRRTSPCRPCPRRSRSRSRSRRKPARKTPPRTCPGACPRPMSPRRGASPRRRKPM
GPFLPTNEIIPGMSPSRVRRIQQKYTTPKRPSTYYDARSRPFKPANDIIPGMSAAEVRRI
KKKYAQPAEESVYFDALDLD

>Shrimp hemocyte iridescent virus isolate 20141215- missing ORF in the annotation of MF599468.1; pI = 11.70
MRVNENICQEFSNRPSVNPLTNRKIQKGKGVYNELKKKCDSLGCRVQSRSRSRSRSRRSS
RRRSSRRRTSPCRPCPRRSRSRSRSRRKPARKTPPRTCPGACPRPMSPRRGASPRRRKPM
GPFLPTNEIIPGMSPSRVRRIQQKYTTPKRPSTYYDARSRPFKPANDIIPGMSAAEVRRI
KKKYAQPAEESVYFDALDLD

Figure S1b. Amino acid sequences of SfAV1a-048R ORF orthologs

Conserved cysteine residues in vs2C-ad motifs are typed and underlined in red, whereas the complete motif is highlighted in yellow. Motifs that are repeated in tandem and rich in arginine-serine residues are highlighted in gray.

>DpTV (previously DpAV4)-ORF008 (one 2-cysteine adaptator and one remnant et the N-term ; pI = 9.29
MALPKLSAAEFATMSAAWEANKMSDPENPTNPLSGRKIKRDGAVWKKVENYFNGATTPVK
SPKGRSPKKEAKPRGRSPKKEAKPRGRSPKKEAKPRARSPKKAISGSSPRREAAAKPAPK
AGAKPTEQDCAEFDRNPGVNPLTSRKIKIDGPVYKKLQKDCADIAPVAGPSGLAGPSGLA
GPSGLGSVGAGSVSVSVPDVVEIDNKVFVDSAANITRQEAFAKLADDRGYYVVKLPETKC
AFTAFGTYFNMTSDEVRNYIVEYATRCGATNPRFVFDDAMIEDIRSGDGDCSRDTLILLS
VAFDCTIRVFYEETKTFELISEGSINRLVQLGLTLDGHYVVMIKTGEQQQILSLPATVTC
SPVVSQRAAPPTPVVSPEDVASMVEALESVRNKPRIYTLLQSEQALLKTIGLI

>HvAV3e-ORF061 (three 2-cysteine adaptator) ; pI = 12.32
MASRRKPSRLTAAQCETFIRNNKAVSPLTNKPIDVYGRAAARFRRDCNLSPPPTKYTSTV
CKKFLANKSVSPYSGRPIKRGAKLYNDLTKHCSGTRSSSPARSPARRRRVIRSPSPNRRS
SSPRRSASPQRRRASPQRRRASPQRRRASPDRSKPAKRTAANADTRPDLCATFSRNESIN
PITGKKLIGTSPIRKAWHRMCAGTPNTRATKCMAFDKNDKKNPFTGRSIRPEQPAYRMVY
SMCHGVPYRSPKRTRRSPVRRSPSPRPYTATSVTRKYRRIKTPARSRSRSRSNSVGRRRT
TAVKSRTKSPARRQSVARSRSRSKSPVRKTTRSRTKSPARRQSVARSRSRTKSPARRQSV
ARSRSRSKSPARRQSVARSRSRSKSPARRQSVAKSRSRSKSPARRQSVAMSRSRSRSRSR
QPMTAMRRSTSRARSRSKSRSRKAMTASRSRSRSVSRYMINPNFVPTTTKKKRVSPVSRG
RINNSRSRSASRARSGGLSPYRGRVLLSPIPDGATPMSRSQLINIANNMNIAELRHIVVS
NGFQPVRVAQNTTQSQLLNIVKFQIREGNLKWLPRNDQDVPTYYTSSRPFADRMKKN

>HvAV3g-ORF066 (three 2-cysteine adaptator) ; pI = 12.33
MASRRKPSRLTAAQCETFIRNNKAVSPLTNKPINVYGRAAARFRRDCNLSPPPTKYTSTV
CKKFLANKSVSPYSGRPIKRGAKLYNDLTKHCSGTRSSSPARSPARRRRVIRSPSPNRRS
SSPRRSASPQRRRASPQRRRASPQRRRASPDRSKPAKRTAANADTRPDLCATFSRNESIN

PITGKKLIGTSPIRKAWHRMCAGTPNTRATKCMAFDKNDKKNPFTGRSIKPEQPAYRMVY
SMCHGVPYRSPKRTRRSPVRRSPSPRPYTATSVTRKYRRIKTPARSRSRSRSNSVGRRRT
TAVKSRTKSPARRQSVARSRSRSKSPVRKTTRSRTKSPARRQSVARSRSRSKSPARRQSV
ARSRSRSKSPARRQSVARSRSRSKSPARRQSVAKSRSRSKSPARRQSVAMSRSRSRSRSR
QPMTAMRRSTSRARSRSKSRSRKAMTASRSRSRSVSRYMINPNFVPTTTKKKRVSPVSRG
RINNSRSRSASRARSGGLSPYRGRVLLSPIPDGATPMSRSQLINIANNMNIAELRHIVVS
NGFQPVRVAQNTTQSQLLNIVKFQIREGNLKWLPRNDQDVPTYYTSSRPFADRMKKN

>SfAV1a-ORF048 (four 2-cysteine adaptator) ; pI = 12.18
MASKRKPARLNAEQCETFKRNKQAVSPLTNCPIDKFGRTAARFRKECDIASPPTRYTSSV
CKKFLANKTVSPYSGRPIKPGKKLYNDLEKHCSGRGTSPSRRSRSRSMSPRRRASPARRR
ASPNRSKPAKRTAANADERPDYCTNFHRDESRNPLTGKKLVPTSPIRKAWHKMCSGTVQT
RSTKCIAFDKNDKINPFTGRPINENNDTYRMIYSMCHGARYLPKKRSPRRKNKSPARTVS
FSPNRRSRSPSIGARRRPARPLRPSTSRSKTRSPSKSRSPSRRRSASKSRSPSRRRSASK
SRSPSRRRSASKSRSPSRRRSASKSRSPSRRRSASKSRSPSMRRSMSMARRSPSQRRSTS
VARRSPSQRRSTSVARRSPSQRRSMSVARRSPSQRRSMSVARRSPSQRRSTSVARRSPSQ
RRSTSVARRSPSQSRRMTTPSRSPSRQRTSSSSRRMSARRSPSYSMSPYRGRVLMTPMPE
DAEPLSQDQLMRIASRMNVAQLRHVVTRNGFQPVDVAFNTNSSQLLNLVRYHIREGNIKW
LPANDQNVPNYRTTARPFMDRMKKN

>TnAV2c-ORF141 (four 2-cysteine adaptator) ; pI = 12.28
MPQATTPRRRRNAQNMNNSAERVTRTQCEDFISSLQKKNPITNRKIDVFGSTAASLRRNC
SMKHNYEFTAPRRSKANMKCTEFLSNPRENPITGRKLAANKPTYKKFVKVCGSPGRISPS
PRRRVTQTRSPSPRRRATQSRSPSPRRRVTQTRSPSPSRRRRSRSRSNSVEFSTNCTKFI
NNDKINPKTNKNIKYKGPTYKKIVTKCKQNLNESPKLPQQMCNEFHQNAKKKNPITGRKI
AVGSVVQRRIVSQCGGGFRSPSRSRSRSASRRRSPSPARSRSRSASRRRSPSPARSRSRS
ASRRRSPSPARSRSRSASRRRSPSPARSKSRSQTRSRSRSTSRRSASPARSKSRSQTKSR
SRSPSPARSRSRSTSRRSASPARSKSRSQTKSRSRSPSPARSRSRSTSRRSASPARSKSR
SKTRSRSRSASKRRSASPARSKSRSQTRSSTRSPSPARSKSRSQTRSRSRSPSSSSSSSR
SRSTSSSRFRSLEQKSLPELRKYAIDLKLSTMGHVYSLDKYSLLRLIKSKEGSTVSKSSS
RSRSSCSSASSKTNCVTGGRRGITDYYSMTVDQLRSLASAKKLGTEEILRNLNAQALRVL
LTSKTPIRLTPQSPNRTFRLQTPQPGTSSTDPLVSKNFGVFKQNRRLL

IIV6-ORF232R (two 2-cysteine adaptator) ; pI = 10.41
MNNNQCMRKKLDELRNIARSYNISITGKKKQQLCDEIIDYQKNNPPRRSPSPRRSPSPRR
ISPECEQWLANKGINPRTGKAIKIGGPTYKKLEMECKEASPKIPSPVRQPSPVHSPVRSP
VRQPSPVRFVEKTKGALNKMKKDQLIDFAQSLGLNPGKLLKPALVDLIFVNQKPPRRSPS
PRRSPSPRRSPSPRRSPSPRPVFVEKTKGALNKMKKDQLIDLAQSLGLNPGKLLKPALVD
LIFVNQKPVEPIRASSSRSSRSTRRSSSTKPSRRSSSRSRRSSSRSRRSSSRSRRSSSR
SRRSSRRSTSRSRSLSKRSIRNISTVGDLEDLVASNLPIAIPESLSRSLSPSRTDFHEAE
IELGSDFDLNNLPENRIAELKQLNVLAKQNGFRMINVPLDGNCMFSVIGRAFNTSSSVIR
QHTVDYLRRCKGSFDHIPANIDDPTINWNDYIDRLEEDACWGDNTALFAASLALNFQAHI
LQVAGGDEGSWIRFGVNETNMGRIVNMGYLDNFHYIALEPFSGRLDILSIPSTHSKCPPP
EISNRRDEEIRRDEEVEDEVIGERIVREAEVIERELRQEEELTSIVSTKRSLRPSIPPKI
STEHRRTPKLRPSVPRPSSIRQSQPNVAALARLETLTKIKDIIDALQRPLENKLSTLTNT
EKAIMQCIGVA

>IIV31-ORF015R (one 2-cysteine adaptator) ; pI = 9.40
MNRNQCMKKKKDELVEIALSMNISVAGKTKEKLCNEILSMQAVPAAPVASPDIASGSDCE

EWIKNPGVNPKTGRAIKIGGPVYKKLEKECGEPEPEEEEEAVVTAIPAIPLYTKGVLNKM
KKNQLIEIALSLSINPDKKLKPKLVEEILIQQTRAVQVPRSPTKLVEEILIQQTRAVQVP
RSPSPRRSPSPRRSPSVVYTKGALNKMKKQDLINLSKTLKLETNGLKQDLIQRILNSYIV
EIEEIAAPRSPSPRRSPSPRRSLSPRRSPRRSPSPSRQSTSVELEISARDVRKISNVSDL
NKLVASIHSPVIRRTSESSFFKNLRRDYSDGKIPRETEFKDLEILAQAAGFKMINVPLDG
ACMFSVIARSFGTTGANIRKIVVDYLRKCEESFAYLFEDYTIPERYLERLEEDDCWGDEL
TLFAATKALNFQAKVLNQHNRQWVDVGSDAGDRIIYLGYIYQFHYVALEKLEKLEGEDET
LILPTSPICPPPMGVSSRSSPRIPSFRPSVPPSIRASVIKSLQPSIPRSLTPAFTPQPTR
LMTLTNIGAIIRELQIPLENKLTTLNDTNTAIMKCVGLA

>Cherax quadricarinatus iridovirus - O57R ; pI = 9.72
MEFNRKRYKDLQIVKSGEILTVPARKISSSPVCIQWFNNPNVNPRTNRKIKTFGPTYRAL
TDECTRKRVRSDPREQRKPPRVTPRVISQPTSNKSWFDIRLRTGIEINNMLREMDVKQWN
LCMSGTKSSKFQKNFTSIEKIGLGSFGQIYKARLSDGNSVVVKEAYLKLPEKRLAEKYTK
KGEKWEDVDVKSYPRENKILELVNQLLLSRKCPNFVYVYNIAFCDGCVIKNYYQRRSTRG
ACYITFMEPADDNLRNTELRTYDQQLSVLYQLLISVHAIHKYYTIWHRDIKSTNIFIKKI
KPGGYFKYVINGKNYFVKNTGIVAYLADFGVSEIMSPLYSSGRYYGTRNGEVAKMDRKIK
GSNLYWKPIYIKGREIRYWYDETSSKNLDYDVIKGTRNKIATKNPIKSSRPIDLNDNHKF
PPFEFGSDIQDVVNVFLGGKQQEQPGNHSRMPYLNSKIRSMLELKAKNTTIVNSIYGTVK
YILADEMLNALYIEPKVVDKIIDTFEMY

> Shrimp hemocyte iridescent virus isolate 20141215 - 94L ; pI = 9.72
MEFNRKRYKDLQIVKSGEILTVPARKISSSPVCIQWFNNPNVNPRTNRKIKTFGPTYRAL
TDECTRKRVRSDPREQRKPPRVTPRVISQPTSNKSWFDIRLRTGIEINNMLREMDVKQWN
LCMSGTKSSKFQKNFTSIEKIGLGSFGQIYKARLSDGNSVVVKEAYLKLPEKRLAEKYTK
KGEKWEDVDVKSYPRENKILELVNQLLLSRKCPNFVYVYNIAFCDGCVIKNYYQRRSTRG
ACYITFMEPADDNLRNTELRTYDQQLSVLYQLLISVHAIHKYYTIWHRDIKSTNIFIKKI
KPGGYFKYVINGKNYFVKNTGIVAYLADFGVSEIMSPLYSSGRYYGTRNGEVAKMDRKIK
GSNLYWKPIYIKGREIRYWYDETSSKNLDYDVIKGTRNKIATKNPIKSSRPIDLNDNHKF
PPFEFGSDIQDVVNVFLGGKQQEQPGNHSRMPYLNSKIRSMLELKAKNTTIVNSIYGTVK
YILADEMLNALYIEPKVVDKIIDTFEMY

>EHNV-ORF089 (Epizootic haematopoietic necrosis virus ; ACO25279.1) ; pI = 9.42. Orthologs in other ranavirus are found in the European catfish virus (YP_006347710.1), the Tiger frog virus (YP_031597.1), the Common midwife toad ranavirus (AFA44994.1), the Soft-shelled turtle iridovirus (ACF42240.1), the Rana grylio iridovirus (AFG73063.1) , the Ambystoma tigrinum virus (YP_003855.1), the Grouper iridovirus (AAV91044.1), and the Singapore grouper iridovirus (YP_164134.1).
MATNYCDEFERNPTRNPRTGRTIKRGGPVFRALERECSDGAARVFPAAAVRGAAAARAVS
PRVAVASPCPEFARDPTRNPRTGRPIKRGGPVFRALERECADYGGASPRRVSPARAFPNR
RASPARRQSPAEAAEASPCPDFARDPTRNPRTGRTIKRGGPTYRALEAECADYGRLSPIR
SPWSDWSSTGSSPFRSHMRKSPAIKSPARKSPARKSPARKSLARYAEYLTSDSETEVDYD
AMNVIRSKVGPGGVCERFAADPTRNPVTGSPLSRNDPLYTDLMEICKGYPDTPLTKSLTG
EGTDDDTCEAFCRDPTRNPVTGQKMRRNGIEYQMFAEECDCSGISRPSGVSRTSGPSGTS
GTSASSRPPNSFEASGVARVPGTPSVSRDEPRWMSSISTRHDYDESNPMSVAFRLRHVKD
IRKFLRTVKPGRSGFCATDNGGWLGSAAVSDKVIGQGSWGSVHMVKFRDFPKEFVVKEAV
LMSVSEKRRYKPTVVWDEWAAGSMPDEVVVNNMVTEIAATGMTPFVPLTAGAGACDSCNP
QLLEKAANVTKCYLQAMEAADFSLDRVLPTMSPDQAASALAQILLGLQSLQTTLGIMHND

IKAHNILVKRVPPGGYWKVTDSFNGQVFYIPNEGYLCMLADYGVVRLVKPAVGMDTLYGT
RNARFVPRDVGRWGKGAGTEYVVTPIRSKISVIVRGGRFVGVEPNKAVRYWKNTDTSKVG
DVITTNNVFYMGYDIEPDMQVQLDDTNSFPIWESRGDVADCVRTFVGGKRASQPGFHGLF
YKKTGSAWEKAAETVAKQNPLFSGFALDGSGLKYIRAATACAYIFPGMAVPRPGEREIES
FTM

Figure S1c. Proteins having the potential to function like SfAV1a P64 protein in some invertebrate iridoviruses (IIV) and members of Lymphocystivirus and Megalocytivirus

Our results provided evidence that the SfAV-1a P64 orthologs found in AV, in some invertebrate iridoviruses and in *ranaviruses* compose a family of large DNA condensing proteins with distinct domains not present in other protein families with a similar function. Its absence in some invertebrate iridoviruses (IIV3, IIV20, IIV22A, IIV25 and IIV30) (Delhon et al., 2006; Piégu et al., 2013) and in vertebrate iridoviruses of the *Lymphocystivirus* and *Megalocytivirus* genera raises questions about which candidate proteins could potentially condense viral DNA for encapsidation in virion. Data presented here, and more specifically those obtained with the rN-term and rC-term domains of SfAV1a P64, provide evidence that the main sequence determinants to bind viral DNA and to assemble DNA-protein aggregates are not the vs2C-ad motifs, but results from the properties of the motifs repeated in tandem and rich in arginine and serine residues that occur both in the N-terminal and the C-terminal region of this protein. In agreement with this conclusion, vs2C-ad motifs are known to be found fused to OTU/A20-like peptidases and S/T protein kinases, proteins that have no DNA binding activities and in which these motifs presumably play a role as adaptors that connect the kinases and OTU/A20 peptidases to their specific targets (Iyer et al. 2006). In SfAV1a P64 and its relatives, the vs2C-ad motifs could therefore be involved in interactions of other virion proteins, including the major capsid protein, with the condensed viral DNA-P64 complex.

In invertebrate iridoviruses, all viruses encoded a protein related to that encoded by the CIV IIV6-178R ORF. This protein met the motif requirements to have a role similar to that of SfAV-1a P64 in condensing viral DNA for encapsidation. To identify candidate proteins that could function in condensing viral DNA for encapsidation in iridoviruses that do not encode a SfAV-1a P64 relative, our searches were focussed on proteins containing vs2C-ad motifs and short tandem repeats rich in arginine and serine residues, with a pI above 9, absent together in AV, IIV3 and *Ranavirus*, and present in all members of the genera *Lymphocystivirus* or *Megalocytivirus*. Among *Lymphocystivirus*, no candidates were found in the Lymphocytis disease virus (Tidona and Arai, 1997). However, we have found in *Megalocytivirus*, a gene family occurring in all virus members and which possesses all criteria, including the difficulty of alignments encountered with the SfAV-1a P64 relatives. This family corresponded to relatives of the Turbot reddish body iridovirus ORF52L.

ORF52L in the Turbot reddish body iridovirus
MPSTTSKCNQLRQNKYTVNPVSNRNIAPRGDTANTLRRICEQPRLCAEYKRSPRYNPWT
DRRLAPGSPKHNLISGMCGGYAPNWSRELVRTNRRAHNTNSRLQREWLETVNRPGAHVP
RLNDACALYYDDPTVNPFTDGPLRRYSPIDDLLYRNCEPAETKRMQCRAFEANPDVNPN
TGRKISPTGPIASSMRRRCMNYDAVPISRSEAGPRGGRSIGVNTPFSANNSNISDTQLS
GSRRSIAVNTPSSHSAHSLLGSISSSSDSPAGPSGVSVGVGPTPGIVIKRSPVRERAE
IIQNYTASRGQQ

ORF055L in the Infectious spleen and kidney necrosis virus
MPSTTSKCNQLRNNRYTVNPVSNRAIAPRGDTANTLRRICEQPRLCAEYKRSPRYNPWT

DRTLAPGSPKHNLISGMCGGYAPNWSRERVRTNRKAHKTNSRLQREWLETVNRPGAHVP
RRDDACALYYDDPTVNPFTDEPLRRYSPIDDLLYRNCESAEIKRRQCRAFEENPDVNPN
TGRRISPTGPIASSMRRRCMNYNAVPISRSEVGPRGGRSIGVNTPFSANNSNISDTQSS
GSHRSIAVNTPSSHSAHSLLGTISSSSSDNSSPAGPSGVSVGVGRTPVALKRSPVRERA
EIIENYTASRGQQ

ORF 106R in the Red seabream iridovirus

MPSTTSKCKQLRRNRYTVNPVSNRSIAPRGDTANTLRRICEQPRLCAEYKRSPRYNPWT
DRTLGPGSPKHNLISGMCGGYAPDWSRERVRTNRKAQNTNSRLQREWLETVNRPGAHVP
RRDDACALYYDDPTVNPFTDGPLRRYSPIDDLLYRNCEPAETKRIQCRAFEENPDVNPN
TGRRISPTGPIASSMRRRCMNYDAMPISRSEVGPRGGRSVGVNTPFSANNSNISDTQFS
GSRRSIAVNTPSSGGSHSAHSLLGSISSSGSDNSSTAGPSGVSVGVGPTPGIVIKRSP
VRERAEIIQNYTASRGQQ

ORF52L in the Turbot reddish body iridovirus

MPSTTSKCNQLRQNKYTVNPVSNRNIAPRGDTANTLRRICEQPRLCAEYKRSPRYNPWT
DRRLAPGSPKHNLISGMCGGYAPNWSRELVRTNRRAHNTNSRLQREWLETVNRPGAHVP
RLNDACALYYDDPTVNPFTDGPLRRYSPIDDLLYRNCEPAETKRMQCRAFEANPDVNPN
TGRKISPTGPIASSMRRRCMNYDAVPISRSEAGPRGGRSIGVNTPFSANNSNISDTQLS
GSRRSIAVNTPSSHSAHSLLGSISSSSSDSPAGPSGVSVGVGPTPGIVIKRSPVRERAE
IIQNYTASRGQQ

References: Supplemental Figure S1c.

Delhon, G., Tulman, E. R., Afonso, C. L., Lu, Z., Becnel, J. J., Moser, B. A., Kutish, G. F. Rock, D. L. 2006. Genome of invertebrate iridescent virus type 3 (mosquito iridescens virus). *J. Virol*. **80**, 8439-8449.

Iyer, L. M., Balaji, S., Koonin, E. V., Aravind, L. 2006. Evolutionary genomics of nucleo-cytoplasmic viruses. *Virus Res.* **111**, 156-184.

Piégu, B., Guizard, S., Spears, T., Cruaud, C., Couloux, A., Bideshi, D. K., Federici, B. A. Bigot, Y. 2013. Complete genome sequence of invertebrate iridescent virus 22 isolated from a blackfly larva. *J. Gen. Virol*. **94**, 2112-2116.

Tidona, C. A., Darai, G. 1997. The complete DNA sequence of lymphocystis disease virus. *Virology*. **230**, 207-216.
